# Changes of ubiquitylated proteins in atrial fibrillation associated with heart valve disease: proteomics in human left atrial appendage tissue

**DOI:** 10.3389/fcvm.2023.1198486

**Published:** 2023-08-28

**Authors:** Chen-Kai Wu, Shuai Teng, Fan Bai, Xiao-Bo Liao, Xin-Min Zhou, Qi-Ming Liu, Yi-Chao Xiao, Sheng-Hua Zhou

**Affiliations:** ^1^ Department of Cardiology, The Second Xiangya Hospital, Central South University, Changsha, China; ^2^ Department of Cardiovascular Surgery, The Second Xiangya Hospital, Central South University, Changsha, China

**Keywords:** atrial fibrillation, ubiquitination, proteomics, protein-protein interaction networks, TTN, MYH6

## Abstract

**Background:**

Correlations between posttranslational modifications and atrial fibrillation (AF) have been demonstrated in recent studies. However, it is still unclear whether and how ubiquitylated proteins relate to AF in the left atrial appendage of patients with AF and valvular heart disease.

**Methods:**

Through LC–MS/MS analyses, we performed a study on tissues from eighteen subjects (9 with sinus rhythm and 9 with AF) who underwent cardiac valvular surgery. Specifically, we explored the ubiquitination profiles of left atrial appendage samples.

**Results:**

In summary, after the quantification ratios for the upregulated and downregulated ubiquitination cutoff values were set at >1.5 and <1:1.5, respectively, a total of 271 sites in 162 proteins exhibiting upregulated ubiquitination and 467 sites in 156 proteins exhibiting downregulated ubiquitination were identified. The ubiquitylated proteins in the AF samples were enriched in proteins associated with ribosomes, hypertrophic cardiomyopathy (HCM), glycolysis, and endocytosis.

**Conclusions:**

Our findings can be used to clarify differences in the ubiquitination levels of ribosome-related and HCM-related proteins, especially titin (TTN) and myosin heavy chain 6 (MYH6), in patients with AF, and therefore, regulating ubiquitination may be a feasible strategy for AF.

## 1. Introduction

One of the major causes of systemic embolism and cardioembolic stroke is atrial fibrillation (AF), which is caused by hemodynamic instability and blood hypercoagulability in clinical practice. Overall, the prevalence of AF will continue to grow with population aging. In the Chinese population over 40 years of age, the prevalence of AF was 1.57% in 2013 (1). AF is still an important public health concern. Drugs and radiofrequency ablation exhibit finite efficiency and safety, and the molecular mechanism underlying the progression of AF remains unclear.

Posttranslational modifications (PTMs) of proteins are covalent modifications. PTMs include ubiquitination, which involves the addition of ubiquitin to a protein, methylation, glycosylation, phosphorylation, acylations, acetylation, and lipidation (2). Ubiquitination is a dynamic multifaceted posttranslational modification where a small protein called ubiquitin is added to a target protein, and ubiquitin, a protein consisting of 76 AAs, is the fundamental unit of ubiquitination. The ubiquitin–proteasome system (UPS) is vital for targeting specific proteins for degradation and controls cellular processes such as protein sorting, signal transduction and DNA repair. Ubiquitination also regulates protein localization and function independent of its effect on protein degradation (3, 4). Ubiquitination plays a central role in cardiovascular diseases (5), such as cardiac hypertrophy (6), congenital heart defects (7), diabetic heart diseases (8–11), and cardiac arrhythmias (12–16), functions with myocardial β 1-adrenergic receptor under physiological conditions (17), and is involved in redox homeostasis, such that it can create an imbalance that leads to cardiovascular complications (18). Furthermore, ubiquitination also plays a role in the occurrence and development of atrial fibrillation. For example, in atrial cardiomyocytes of rabbits with atrial fibrillation, Rfp2 has been found to be upregulated, which leads to the ubiquitination of Cav1.2 to autophagosomes, and these changes induce atrial electrical remodeling (19). Another recent study clarified the relationship between the E3 ubiquitin protein ligase tripartite motif-containing protein 21 and atrial remodeling and AF after myocardial infarction atrial remodeling (20). The degradation of Smad7 by Arkadia-mediated polyubiquitination plays an important role in AF-induced atrial fibrosis (21). Although several studies report evaluations of ubiquitination in the context of AF, proteome-wide analyses have not yet been conducted. The purpose of this study is to clarify whether there are changes in ubiquitination during atrial fibrillation, what specific alterations occur, and how they relate to functional changes.

In this study, we performed LC–MS/MS analysis to identify proteins with ubiquitination that were altered in AF tissues. A total of 271 sites in 162 proteins exhibiting upregulated ubiquitination and 467 sites in 156 proteins exhibiting downregulated ubiquitination were identified. Most upregulated and downregulated ubiquitylated proteins were located in the cytoplasm. A protein–protein interaction (PPI) network analysis revealed that glycolysis-related, ribosome-related, endocytosis-related, and hypertrophic cardiomyopathy-related proteins were highly associated with ubiquitination in AF tissues.

## 2. Subjects and methods

### 2.1. Study design

A single-center study was conducted with patients undergoing cardiac valvular replacement surgery at the Second Xiangya Hospital of Central South University. Based on their symptoms and results of 12-channel electrocardiography or 24-h Holter electrocardiography, the subjects were assigned to a chronic AF group (onset time ≥1 year) or a sinus rhythm (SR) group. All patients underwent transthoracic echocardiography (TTE) before surgery, and other general patient data were collected. Based on coronary angiography (CAG), patients with coronary artery disease (CAD) were excluded from the study. A piece of tissue was sliced from the left atrial appendages (LAA) of the enrolled patients during surgery.

**Figure 1:**
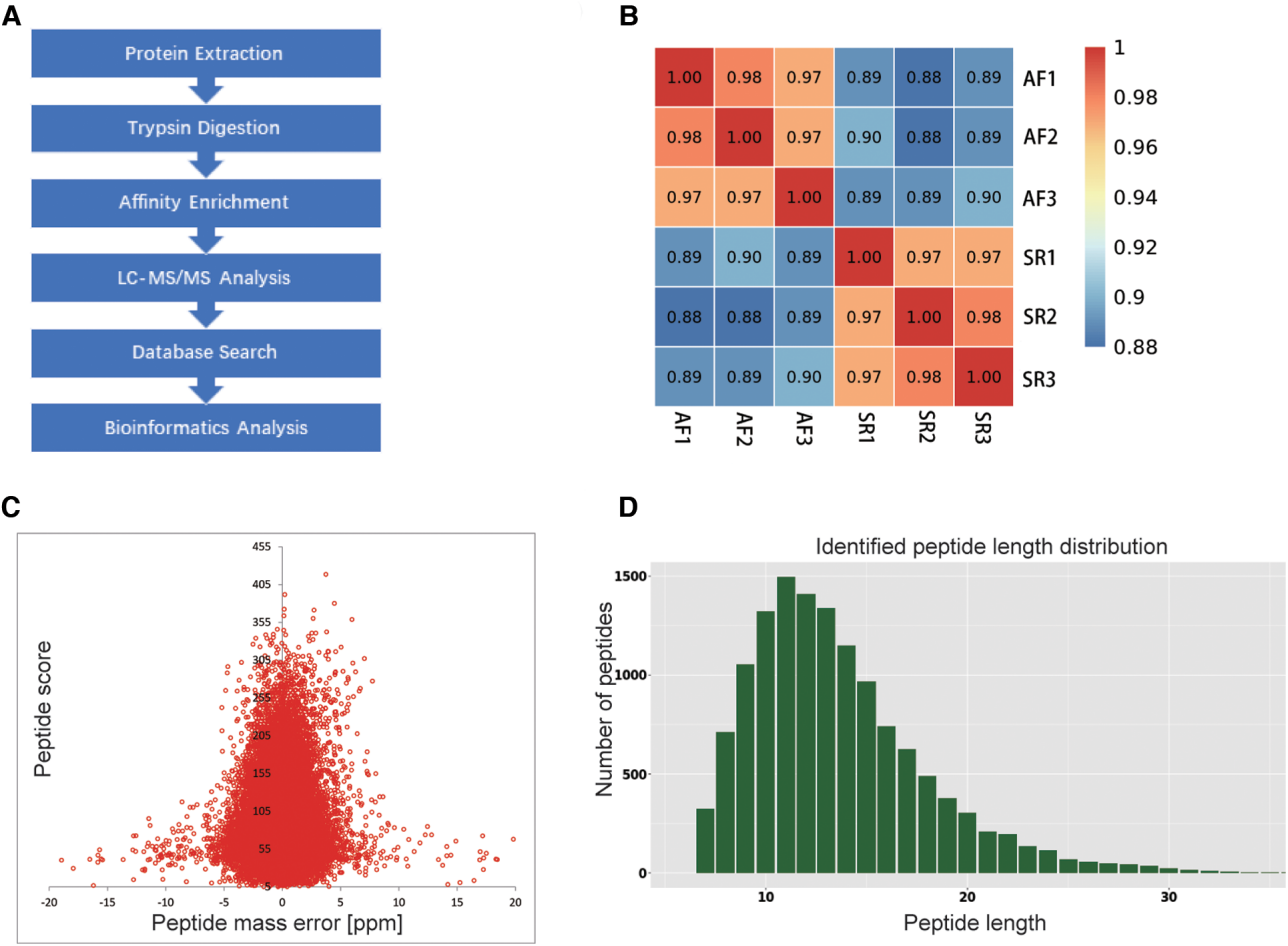
Flow chart of the study and QC validation. (**A**) Flow chart showing the proteomic ubiquitination analysis of SR and AF samples. (**B**) Consistency among 3 mixed tissue sample replicates was indicated by Pearson's correlation coefficient. (**C**) QC validation of the MS data: the accuracy of the mass data was in line with that needed, and most mass errors were <5 ppm. (**D**) The length of most peptides ranged from 7 to 20 AAs.

We obtained ethical approval for the study from the Ethics Committee of the Second Xiangya Hospital of Central South University. Written informed consent was obtained by all the subjects or their legal representatives. The study was conducted based on the Declaration of Helsinki. The flow chart of the study is illustrated in Figure 1A.

### 2.2. Protein extraction and trypsin digestion

The specimens were obtained from the LAA during mitral valve surgery and were immediately flash-frozen in liquid nitrogen and stored in liquid nitrogen. To analyze the ubiquitination characteristics of the samples, a mixture of 3 tissue samples from the same group was considered to be one sample. We obtained 3 of these mixed samples for each group. The ground tissue was lysed by sonication in lysis buffer. Debris was removed through centrifugation. Finally, a BCA kit was used to measure the protein concentration of the prepared samples.

Equal amounts of protein were taken based on concentration measurement, and the volume was adjusted with lysis buffer. The samples were then treated with 20% TCA and incubated at 4°C for 2 h. After centrifugation at 4,500 × *g* for 5 min at 4°C, the supernatant was discarded, and the protein pellet was washed three times with cold acetone. The protein pellet was air-dried and then resuspended in 200 mM TEAB followed by sonication to disperse the precipitate. Trypsin was added at a ratio of 1:50 and incubated overnight at 37°C. Dithiothreitol (DTT) was subsequently added to a final concentration of 5 mM and incubated at 56°C for 30 min, followed by alkylation with IAA at a final concentration of 11 mM at room temperature in the dark for 15 min. After that, trypsin was added again at a ratio of 1:100, and the samples were further digested for 4 h.

### 2.3. Affinity enrichment

The peptides obtained were dissolved in IP buffer solution, and the supernatant was poured into a flask containing prewashed antibody-loaded beads, placed on a rotating shaker at 4°C, and gently shaken overnight. After incubation, the beads were washed 4 times with IP buffer and twice with deionized water. Finally, 0.1% trifluoroacetic acid eluent was used 3 times to elute the bead-bound peptides. Finally, the eluted fractions were combined and vacuum-dried. After drying, the resulting peptides were desalted and used for LC–MS/MS analysis.

### 2.4. LC–MS/MS analysis

The peptides were dissolved and then separated using NanoElute Ultra Performance Liquid Chromatography (UPLC). Solvent A was an aqueous solution containing 0.1% formic acid and 2% acetonitrile; solvent B was an acetonitrile solution containing 0.1% formic acid. The gradient consisted of solvent B increasing from 6% to 22% for 43 min, from 22% to 30% for 13 min, and from 30% to 80% for 3 min, all at a constant flow rate of 450 nl/min.

The peptides were separated by UPLC, injected into the NSI source for ionization and then analyzed by a tims-TOF Pro mass spectrometer. The electrospray voltage was set at 1.6 kV, and the peptide precursor ions and their secondary fragments were detected and analyzed by TOF. The m/z scan range of the secondary mass spectrum was set to 100–1,700 m/z, and intact peptides were detected in the Orbitrap at a resolution of 70,000. The data acquisition mode applied was parallel accumulation and serial fragmentation (PASEF). After the first-level mass spectra were collected, the second-level spectra with precursor ion charges in the range of 0–5 were collected in PASEF mode 10 times, and the dynamic rejection time applied to the tandem mass spectrum scan was 24 s to avoid repeated scans of the precursor ions. The concentration of the injection was 2 mg, the analysis temperature was set as 50°C, and the runtime of analysis was set as 60 min. We used a homemade reversed-phase analytical column (100 μm i.d. × 25 cm) packed with 1.9 μm/120 Å ReproSil PurC18 resins (Dr. Maisch GmbH, Ammerbuch, Germany).

### 2.5. Database search

Secondary mass spectrum data were searched using MaxQuant (v1.6.6.0). The following search parameter settings were used: The database was Homo_sapiens_9606_SP_20191115 (20380 sequences), a reverse database was applied to calculate the false-positive rate (FDR) caused by random matching, and a common contamination library was applied to eliminate the impact of contaminating proteins on the identification results; the cleavage enzyme was set to Trypsin/P. The FDR for protein identification and peptide-spectrum match (PSM) identification was adjusted to <1%.

### 2.6. Bioinformatics methods

The UniProt-GOA database (http://www.ebi.ac.uk/GOA/) was used for Gene Ontology (GO) annotation of the proteome. GO covers three domains: cellular component, molecular function, and biological process. Kyoto Encyclopedia of Genes and Genomes (KEGG) pathway annotation was derived from the KEGG database (http://www.genome.jp/kegg/). The subcellular localization prediction software WoLF PSORT (https://wolfpsort.hgc.jp/) was used. The motif characteristics of the ubiquitylated AAs were analyzed using MoMo software (the motif-x algorithm). A protein–protein interaction enrichment analysis was performed with the STRING database version 11.0. A corrected *P value* <0.05 was considered statistically significant in the bioinformatics analysis. The methodology used for the validation analysis was a two-sample *t* test.

### 2.7. Data availability

The primary proteomics data produced in this study are uploaded to PRIDE: Project PXD030397 https://www.ebi.ac.uk/pride/archive/projects/PXD030397.

## 3. Results

### 3.1. Subject characteristics

Table 1 summarizes the demographic and baseline characteristics of the patients. The parameters in the SR and AF groups were matched with respect to gender ratio, BMI (25.25 ± 5.09 vs. 22.33 ± 3.33 kg/m^2^, *P* = 0.169), age (59.22 ± 6.72 vs. 56.44 ± 9.26 years, *P* = 0.477), fasting blood glucose (4.81 ± 072 vs. 5.03 ± 0.69 mmol/L, *P* = 0.493), triglycerides levels (1.34 ± 0.56 vs. 1.06 ± 0.54 mmol/L, *P* = 0.308), total cholesterol levels (4.08 ± 0.70 vs. 3.81 ± 0.27 mmol/L, *P* = 0.303), EF (64.22 ± 9.51% vs. 59.44 ± 7.76%, *P* = 0.260), comorbidities and pharmacological treatment. The difference observed in LA size was significant (35.00 ± 5.92 vs. 59.44 ± 7.76 mm, *P* = 0.000).

**Table 1:** Demographic and baseline data of the subjects.

	SR (*n* = 9)	AF (*n* = 9)	*P* value
Male/female (*n*, %)	3 (33.3%)/6 (66.6%)	5 (55.5%)/4 (44.4%)	0.343
BMI (kg/m^2^)	25.25 ± 5.09	22.33 ± 3.33	0.169
Age (year)	59.22 ± 6.72	56.44 ± 9.26	0.477
Fasting blood glucose (mmol/L)	4.81 ± 072	5.03 ± 0.69	0.493
Triglycerides (mmol/L)	1.34 ± 0.56	1.06 ± 0.54	0.308
Total cholesterol (mmol/L)	4.08 ± 0.70	3.81 ± 0.27	0.303
LA size (mm)	35.00 ± 5.92	58.89 ± 8.37	0.000
EF (%)	64.22 ± 9.51	59.44 ± 7.76	0.260
Mitral valve area (mm^2^)	137.00 ± 26.42	141.00 ± 51.25	0.282
Mitral regurgitation			0.653
Grade II (*n*)	6	5	
Grade III (*n*)	3	4	
NYHA class (II/III) (*n*)	2/7	1/8	0.555
Comorbidities			
Hypertension (*n*, %)	2 (22.2%)	2 (22.2%)	1.000
Congestive heart failure (*n*, %)	9 (100%)	9 (100%)	1.000
Diabetes (*n*, %)	2 (22.2%)	3 (33.3%)	0.599
Stroke or TIA (*n*, %)	0 (0.00%)	0 (0.00%)	1.000
Pharmacological treatment			
Beta blockers (*n*, %)	8 (88.88%)	8 (88.88%)	1.000
ACE inhibitors (*n*, %)	6 (66.66%)	8 (88.88%)	0.257
Amiodarone (*n*, %)	0 (0.00%)	2 (0.00%)	0.134

### 3.2. Quantitative analysis of lysine ubiquitination in LAA

**Figure 2:**
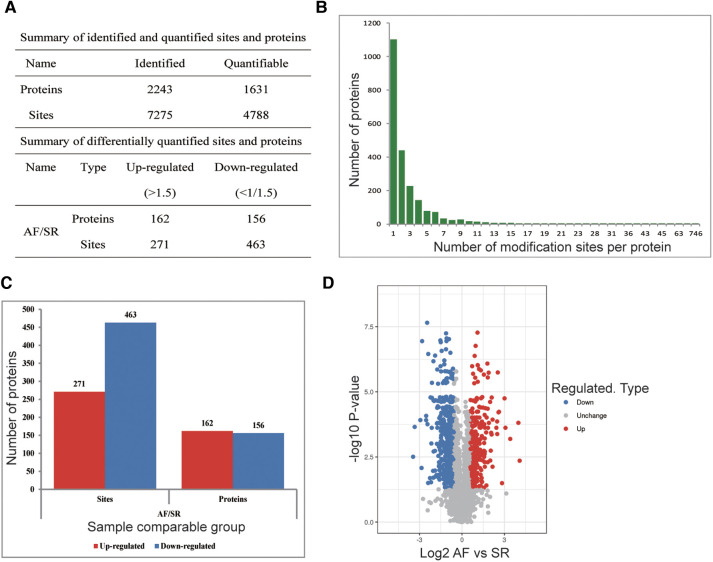
Ubiquitination analysis of SR and AF tissue samples. (**A**) Changes were observed at 4,788 quantifiable sites and in 1,631 quantifiable proteins. (**B**) Identified protein site number distribution: most proteins harbored <5 modification sites. (**C**) Differential ubiquitination sites and protein numbers: A total of 271 sites in 162 proteins exhibiting upregulated ubiquitination and 467 sites in 156 proteins exhibiting downregulated ubiquitination were identified. (**D**) Volcano plot showing differentially ubiquitylated proteins and sites.

To explore whether the ubiquitination rate was influenced by AF status. A quantitative ubiquitination analysis was performed based on UPLC–MS/MS data. A mixture of every 3 tissues from the same group was recognized as one sample. Consistency in the analysis of 3 mixed-tissue samples (triplicate samples) was indicated through Pearson's correlation analysis (Figure 1B). The overall accuracy of the mass data acquired by MS was in line with that required for the analysis, with most mass errors <5 ppm (Figure 1C). As illustrated in Figure 1D, most peptide lengths ranged from 7 to 20 AAs. In general, as illustrated in Figure 2A, changes in ubiquitination were observed in 4,788 quantifiable sites and 1,631 quantifiable proteins. There were <5 modified sites in most proteins (Figure 2B). The quantification ratios for the downregulated and upregulated ubiquitination cutoff values were set at <1:1.5 and >1.5, respectively; 156 downregulated ubiquitinated proteins and 162 upregulated ubiquitinated proteins were identified. As illustrated in Figures 2C,D, ubiquitination was downregulated at 467 sites, and ubiquitination was upregulated at 271 sites.

Among these differentially ubiquitylated proteins, titin (TTN), contributing to sarcomere assembly and conferring stability during the cardiac cycle, harbored the most sites (174). Myosin heavy chain 6 (MYH6) harbored 38 sites, and myosin heavy chain 7 (MYH7) harbored 11 sites. Mutations in MYH6 and MYH7 are associated with hypertrophic cardiomyopathy. MYH6 and MYH7 are the alpha heavy chain subunit and beta (or slow) heavy chain subunit of cardiac myosin, respectively. Myomesin 1 (MYOM1), myomesin 3 (MYOM3) and myomesin 2 (MYOM2) harbored 14 ubiquitination sites, 12 ubiquitination sites and 7 ubiquitination sites, respectively. MYOM1, MYOM2 and MYOM3 are highly expressed in the heart. MYOM1 and MYOM2, parts of an M-band, bind tightly to TTN in the M-line; in addition, MYOM3 is a recently discovered part of the M-band (22). The number of ubiquitination sites in the remaining proteins was as follows: 9 in HSPA1B, 8 in GJA1, 8 in DSP, 7 in HSPA8, 6 in TPM1 and 6 in TUBA1B (Table 2).

**Table 2:** Summary of the 11 proteins with the greatest difference in ubiquitination.

Protein accession	Gene name	Number of sites	Upregulated sites	Downregulated sites
Q8 WZ42	TTN	174	5	169
P13533	MYH6	38	34	4
P52179	MYOM1	14	0	14
Q5VTT5	MYOM3	12	0	12
P12883	MYH7	11	2	9
P0DMV9	HSPA1B	9	9	0
P17302	GJA1	8	8	0
P15924	DSP	8	1	7
P11142	HSPA8	7	7	0
P54296	MYOM2	7	1	6
P09493	TPM1	6	1	5
P68363	TUBA1B	6	6	0

### 3.3. Subcellular location analysis of differentially expressed ubiquitylated proteins

The subcellular localization of the differentially expressed proteins (DEGs) is presented in Supplementary Figure S1. The proteins exhibiting upregulated ubiquitination were mainly in the cytoplasm (*n* = 69, 42.86%), nucleus (*n* = 36, 22.36%) and plasma membrane (*n* = 23, 14.29%). The others were in mitochondria, the extracellular space and other locations. The proteins exhibiting downregulated ubiquitination were mainly in the cytoplasm (*n* = 69, 44.52%), nucleus (*n* = 42, 27.1%), extracellular space (*n* = 12, 7.74%) and mitochondria (*n* = 11, 7.1%).

### 3.4. GO enrichment-based functional classification of differentially expressed ubiquitylated proteins

**Figure 3:**
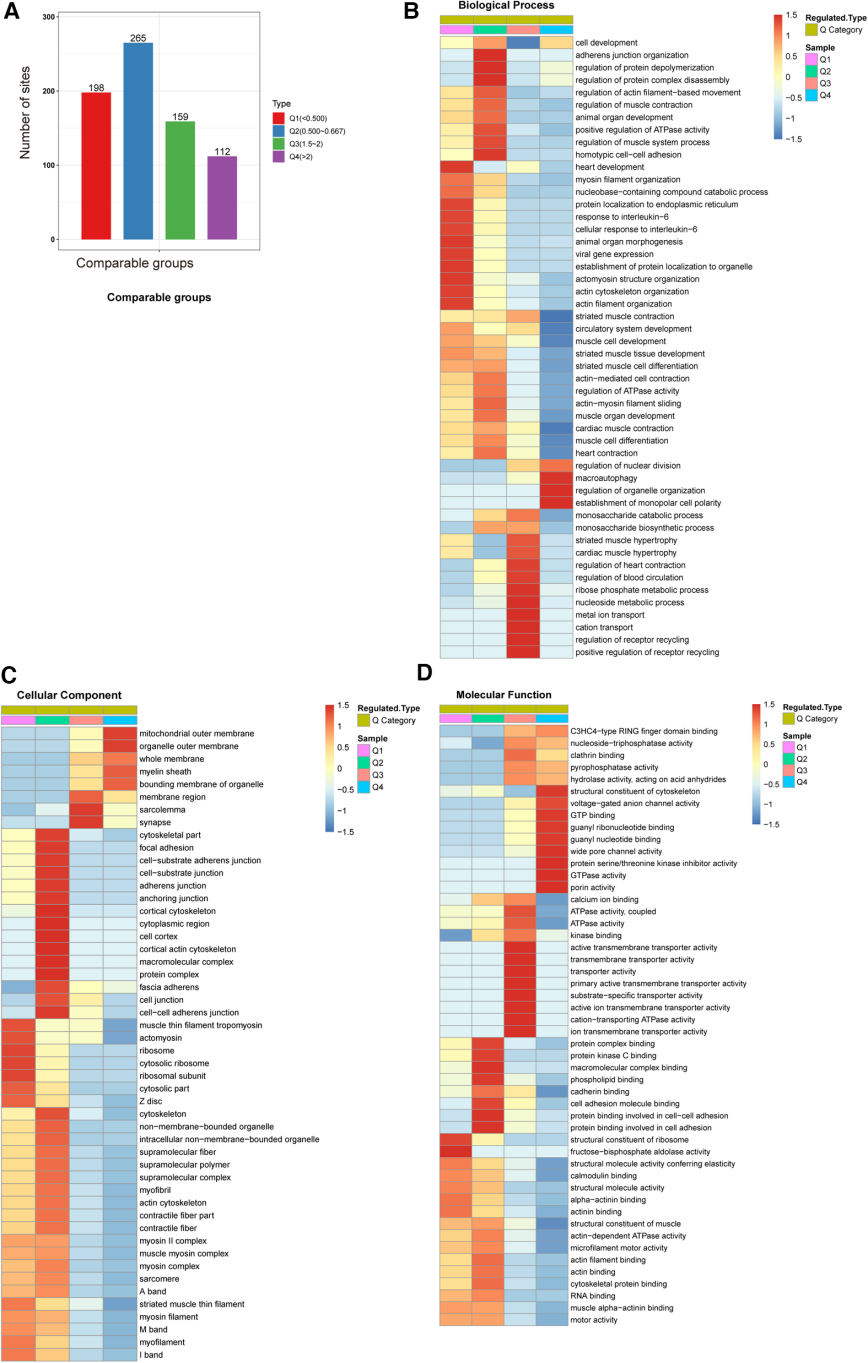
GO enrichment-based functional classification of differentially ubiquitylated proteins. (**A**) We classified the sites with different modification abundances into 4 quartiles, called Q1–Q4, according to their different modification multiples. (**B**) Biological process analysis based on Q1–Q4 quantiles. (**C**) Cellular component analysis based on Q1–Q4 quantiles. (**D**) Molecular function analysis based on Q1–Q4 quantiles.

We classified sites with different modification abundances into 4 quantiles according to their AF/SR modification multiples; these quantiles were called Q1–Q4 (Q1 < 0.500, Q2: 0.500–0.667, Q3: 1.5–2.0, and Q4 > 2.0) (Figure 3A). As illustrated in Figures 3B–D, GO enrichment-based functional classification indicated a relationship among the sites in the same quantile. Figure 3B shows the ubiquitination of sites associated with AF. The results revealed that the downregulated proteins with downregulated ubiquitination in Q1 were mainly involved in processes related to heart development, protein localization to organelles, viral gene expression, protein localization to the endoplasmic reticulum, the response to interleukin-6, animal organ morphogenesis, actomyosin structure organization, and actin filament organization. It also revealed that the ubiquitylated proteins in Q3 were mainly involved in processes related to heart contraction, blood circulation, ribose phosphate metabolism, nucleoside metabolism, metal ion transport, cation transport, and receptor recycling. The proteins with upregulated ubiquitination in Q4 were enriched mainly in macroautophagy, the regulation of organelle organization, and the establishment of monopolar cell polarity.

In the cellular component category, the distributions of proteins with downregulated ubiquitination in Q1 were ribosomes, cytosolic ribosomes and ribosomal subunits. In contrast, proteins in Q3 exhibiting upregulated ubiquitination were highly enriched in sarcolemma and synapses. Q4 proteins were involved in organelle outer membranes and the mitochondrial outer membrane (Figure 3C).

Ubiquitination rates were also analyzed based on molecular functions (Figure 3D). In Q1, the proteins with downregulated ubiquitination exhibited fructose-bisphosphate aldolase activity. The proteins in Q3 exhibiting upregulated ubiquitination were mainly involved in ion transmembrane transporter activity, cation-transporting ATPase activity and transmembrane transporter activity. The Q4 proteins were closely associated with structural constituents of the cytoskeleton, GTPase activity GTP binding, guanyl binding, wide pore channel activity, and porin activity.

### 3.5. Protein domain and KEGG pathway analysis of differentially expressed ubiquitylated proteins

**Figure 4:**
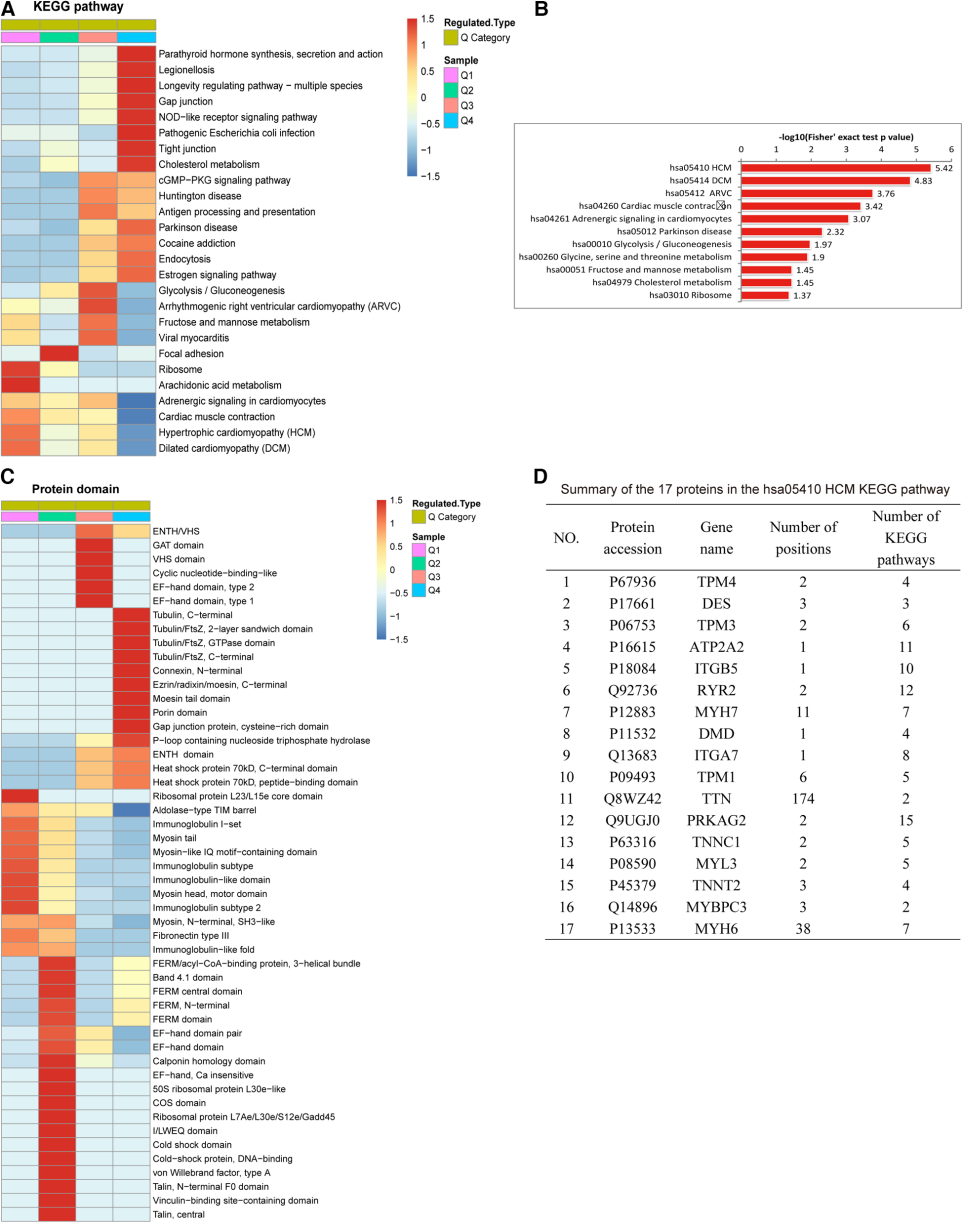
Protein domain and KEGG pathway analysis of differentially expressed ubiquitylated proteins. (**A**) KEGG pathway analysis based on Q1-Q4 quantiles. (**B**) Top 11 pathways of all KEGG pathways. The proteins involved in all KEGG pathways were most highly enriched in hsa05410 hypertrophic cardiomyopathy (HCM). (**C**) Protein complex analysis. (**D**) Summary of the 17 proteins associated with hsa05410 HCM: TTN had the most (348) modifications in all enriched KEGG pathways, followed by MYH6 (266).

Ubiquitination rates were also analyzed based on protein domain analysis. In Q1, the proteins were distributed in the ribosomal protein L23/L15e core domain. In Q3, the proteins were enriched in ATPase, GAT, VHS, cyclic nucleotide-binding-like and EF-hand domains. The proteins with upregulated ubiquitination in Q4 were involved in the tubulin, connexin, ezrin/radixin/moesin, moesin tail, and porin domains, gap junction proteins, and P-loop-containing nucleoside triphosphate hydrolase (Figure 4C).

Figure 4A reveals the results of the KEGG pathway analysis of ubiquitylated proteins associated with AF. In the Q1 quantile, the proteins with downregulated ubiquitination were distributed mainly in pathways related to ribosome and arachidonic acid metabolism. In Q3, the proteins with upregulated ubiquitination were distributed mainly in pathways related to arrhythmogenic right ventricular cardiomyopathy. The Q4-enriched proteins were involved in parathyroid hormone synthesis, secretion and action, legionellosis, pathogenic *Escherichia coli* infection, the NOD-like receptor signaling pathway, longevity regulating pathway, gap junctions, tight junctions, and cholesterol metabolism. Figure 4B reveals that in all KEGG pathways, the proteins were highly enriched in hsa05410 hypertrophic cardiomyopathy (HCM). Among the 17 proteins associated with the hsa05410 hypertrophic cardiomyopathy (HCM) KEGG pathway, TTN harbored the highest number of modification sites (174), followed by MYH6 (with 38) (Figure 4D).

### 3.6. Motif analysis of differentially altered ubiquitylated proteins

**Figure 5:**
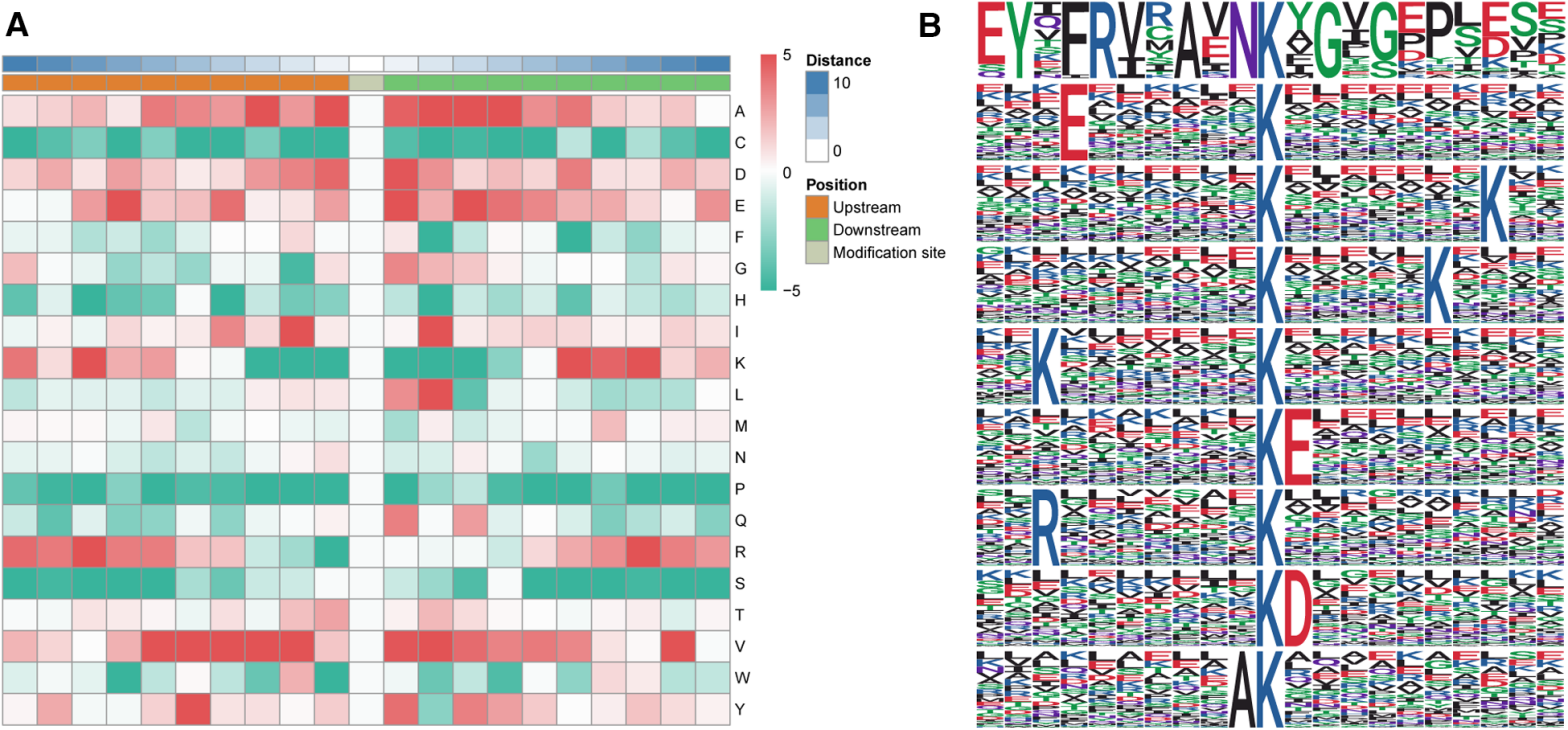
Motif analysis of differentially altered ubiquitylated proteins. (**A**) Heatmap of the 10 bilateral AAs down- and upstream of the ubiquitylated lysine. Enrichments of valine (V), glutamic acid (E), aspartic acid (D) and alanine (A) residues were found downstream of the ubiquitylated lysine, whereas enrichments of valine (V) and alanine (A) were found upstream of the ubiquitylated lysine. (**B**) Sequence logo of acetylation motifs. The results revealed nine significantly enriched ubiquitination site motifs from the quantifiable ubiquitylated sites, where x stands for a random AA and Kac stands for a ubiquitylated lysine.

Based on MoMo software (the motif-x algorithm), a motif analysis of differentially ubiquitylated proteins was conducted to evaluate the 10 bilateral AAs down- and upstream of a ubiquitylated lysine (Figures 5A,B). Sequences enriched with valine (V), glutamic acid (E), aspartic acid (D) and alanine (A) residues were found downstream of a ubiquitylated lysine, whereas sequences enriched with valine (V) and alanine (A) residues were found upstream. Among these motifs, A was at the +4/+3/+2 positions, and L was at the +2 position (Figure 5A).

The results revealed nine significantly enriched ubiquitination site motifs among the quantifiable ubiquitylated sites: YxxRxxAxNKacxG, ExxxxxxKac, KacxxxxxxxK, KacxxxxxK, KxxxxxxxKac, KacE, RxxxxxxxKac, KacD, and AKac (Figure 5B), where x stands for a random AA and Kac stands for a ubiquitylated lysine.

### 3.7. PPI network analysis of differentially ubiquitylated proteins

**Figure 6:**
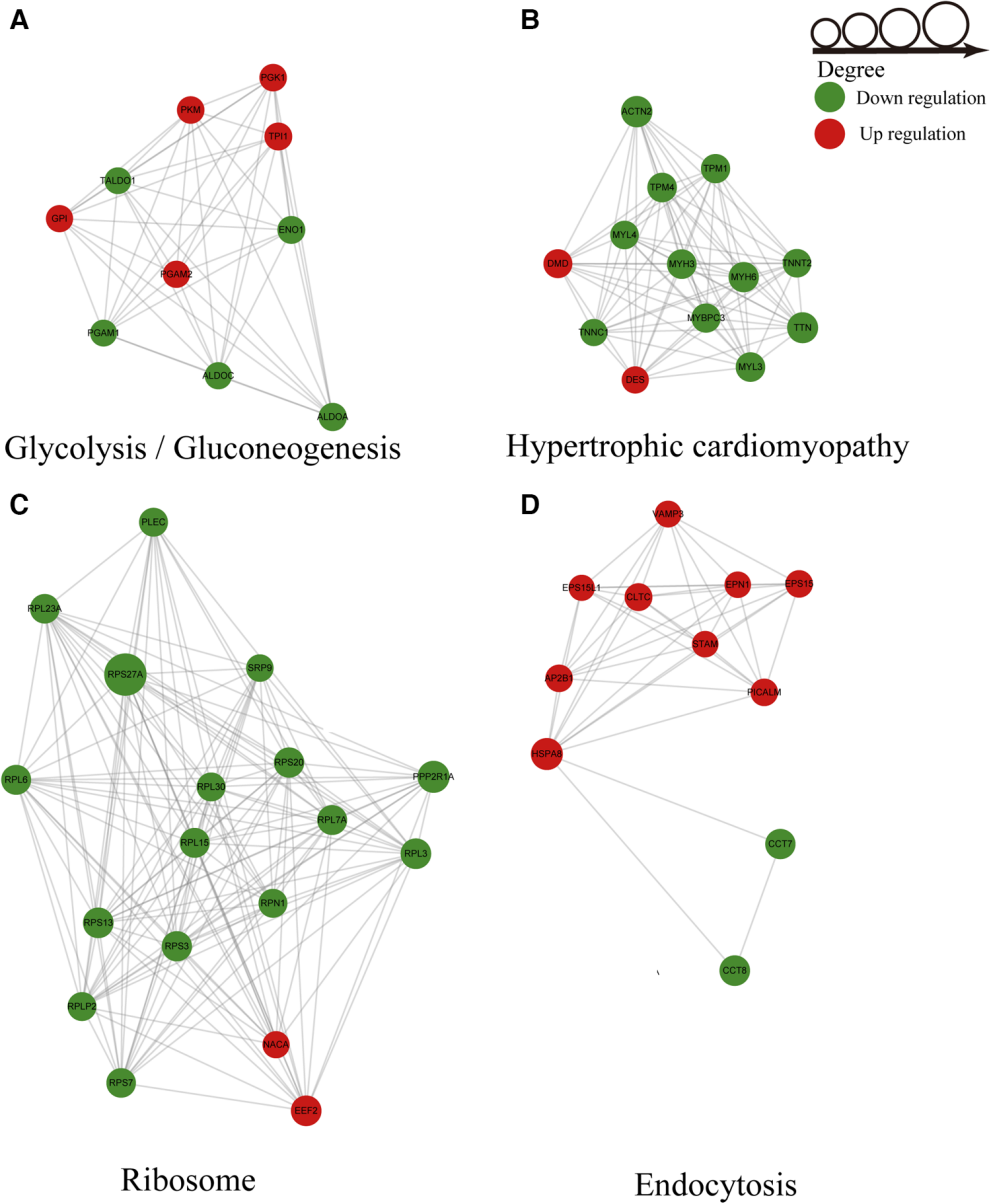
Ubiquitylated PPI network analysis. (**A**) PPI of glycolysis-related proteins. (**B**) PPI of hypertrophic cardiomyopathy-related proteins. (**C**) PPI of ribosome-related proteins. (**D**) PPI of endocytosis-related proteins.

Based on PPI networks and the STRING database, we analyzed the key genes associated with AF. The results revealed that ubiquitylated proteins in AF were mainly glycolysis-, ribosome-, endocytosis-, and hypertrophic cardiomyopathy-related proteins (Figures 6A–D). The ubiquitylated proteins associated with glycolysis harbored five upregulated ubiquitination sites and five downregulated ubiquitination sites (Figure 6A). The HCM-related sites included two sites with upregulated modification and eleven sites exhibiting downregulated modifications (Figure 6B). Sixteen sites in the ribosome-related ubiquitination PPI network showed downregulated ubiquitination, and two sites showed upregulated ubiquitination (Figure 6C). The PPI network associated with endocytosis-related proteins included 9 upregulated ubiquitination sites and two downregulated ubiquitination sites (Figure 6D). MYH6 and TTN were identified as candidate key genes highly related to AF. The cluster identification assay shown in Figure 6C suggested that RPS27A played the most important role in AF.

### 3.8. Validation results

**Figure 7:**
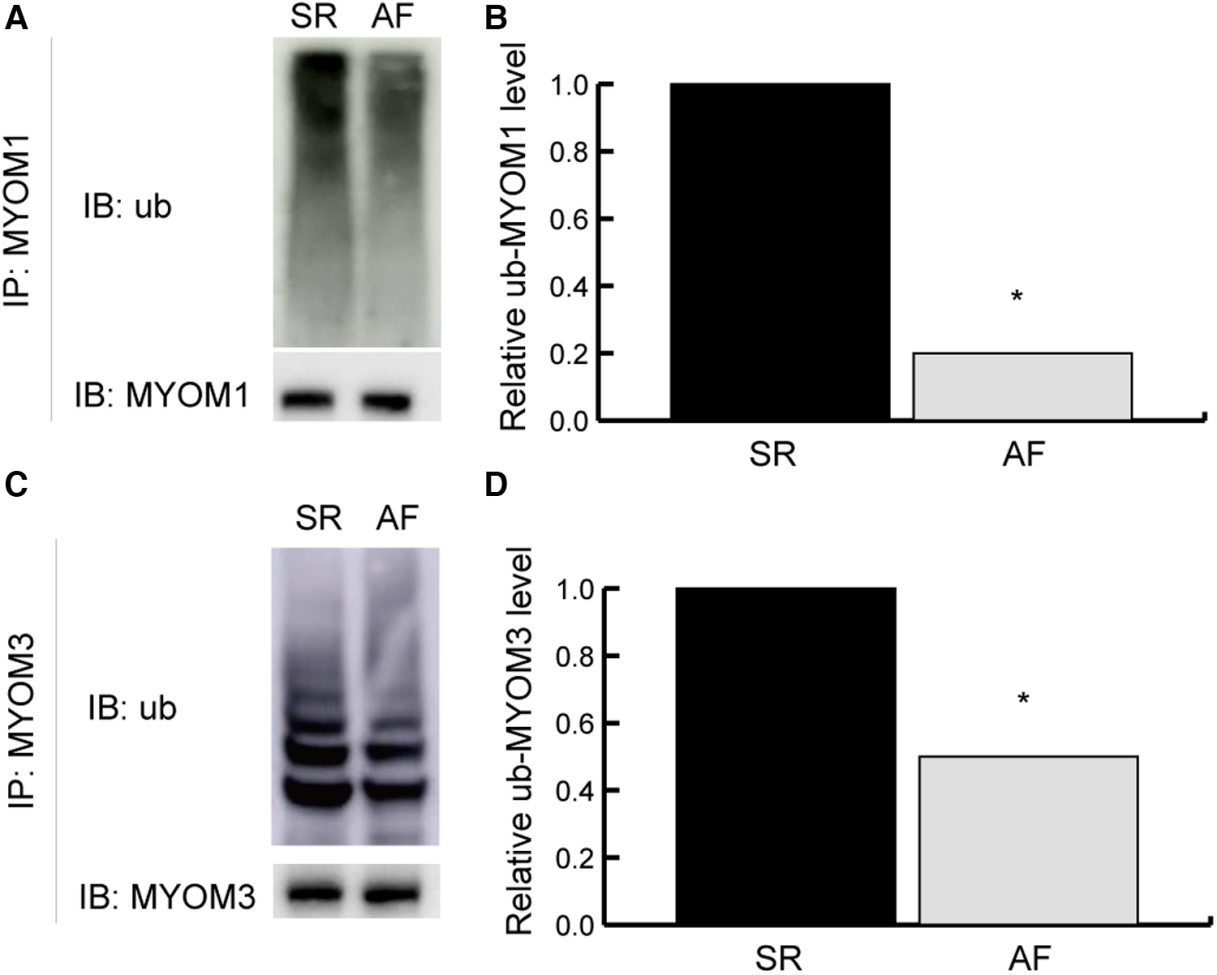
Validation results. Validation of ubiquitylated MYOM1 and ubiquitylated MYOM3 as determined by coimmunoprecipitation (co-IP) combined with Western blotting (WB). (**A,B**) Ubiquitinated MYOM1. (**C,D**) Ubiquitinated MYOM3. IP, immunoprecipitation; IB, immunoblotting; MYOM1, myomesin 1; MYOM3, myomesin 3; ub, ubiquitylated. *P < 0.05.

The identity of myomesin 1 (MYOM1) and myomesin 3 (MYOM3) was validated by immunoprecipitation (IP) combined with Western blotting (WB). MYOM1 and MYOM3 in the LAA tissues from the SR group were preferentially coimmunoprecipitated with ubiquitin compared with those from the AF group (both *P* < 0.05), confirming the differential interactions identified in the mass spectrometry analysis (Figure 7), which demonstrated the reliability of the results in this study (Table 2).

## 4. Discussion

To date, this report is the only description of protein ubiquitination in AF patients based on quantitative proteomics. Our results revealed that the degree of protein ubiquitination was different in AF tissues than in non-AF tissues, leading to upregulated or downregulated ubiquitination. We also identified ubiquitination sites. A total of 271 sites in 162 proteins exhibiting upregulated ubiquitination and 467 sites in 156 proteins exhibiting downregulated ubiquitination were identified. Interestingly, both upregulated lysine ubiquitination and downregulated lysine ubiquitination were identified in some proteins. Furthermore, the DEGs involved in ubiquitination events encoded proteins associated with glycolysis, ribosomes, endocytosis, and hypertrophic cardiomyopathy. These results revealed that ubiquitination is essential to the development of AF.

PTMs are indispensable in numerous cellular processes. Among twenty AAs, modifications are deposited mainly on a lysine residue, the only AA with a side chain amine, which can be covalently modified by glycosyl, propionyl, butyryl, acetyl, hydroxy, crotonyl, ubiquitin and ubiquitin-like groups. Ubiquitination is vital for myocardial ischemia/reperfusion (I/R) injury, cardiomyopathy, and heart failure (23). It has been reported that parkin, cooperating with the ubiquitin-conjugating enzyme UbcH7, functions as a ubiquitin ligase to promote protein degradation (24). Shimura H and his colleague identified a new mechanism that can alleviate myocardial injury. Mitochondrial permeability transition pore (mPTP) opening was suppressed by parkin through the catalyzed ubiquitination of CypD, which is involved in necrotic cascades. Thus, parkin benefits cardiac function (25). The results obtained by Tsushima et al. described a novel mechanism underlying lipotoxic cardiomyopathy. An increasing rate of A-kinase anchor protein 121 (AKAP121) ubiquitination changed the phosphorylation rate of Ser637 in dynamin-related protein 1 (DRP1), which was caused by altered mitochondrial redox status (26). Furthermore, ubiquitination has been associated with ubiquitination in myocardial infarction (MI). Phosphorylation of GSK3 was necessary for the ubiquitination-dependent degradation of OMA1, which promoted leptin-regulated mitochondrial integrity. Leptin enhances the survival and mitochondrial integrity of hMSCs. Therefore, enforcing ubiquitination may be a feasible strategy against cardiovascular diseases such as MI (27).

Our own bioinformatics analysis showed that TTN harbored the most ubiquitination sites (with 174 sites), followed by MYH6, which harbored 38 sites. In addition, TTN harbored the most modified sites (348) in all the enriched KEGG pathways, followed by MYH6 (266). Therefore, the TTN and MYH6 genes were significantly and highly expressed in AF compared with SR tissues. We speculated that these two highly expressed genes may play a role in the incidence of AF in valvular heart disease patients. There have been several studies on the correlation between AF and these two highly expressed genes, TTN and MYH6.

TTN, the largest known protein [*M*(*r*) 3,000 kDa], plays an important role in sarcomere assembly and confers stability during the cardiac cycle (28). A study by Choi et al. indicated a close correlation between early-onset AF and a loss-of-function (LOF) TTN variant (29). In another study, Ahlberg et al. revealed that early-onset AF was closely associated with titin truncation variants (TTNtvs) (30, 31). Furthermore, Chalazan et al. indicated that in ethnic minority groups, there was a close correlation between early-onset AF and TTN variants (32). In addition, TTN has been correlated with other diseases, such as dilated cardiomyopathy, hypertrophic cardiomyopathy, and neuromuscular disorders (33–35). The underlying mechanisms by which TTN leads to AF remain unclear. One possible mechanistic explanation for the highly expressed TTN association with AF is that DCM or HCM caused by TTN increases patient susceptibility to AF. However, none of the patients recruited in this study suffered from DCM or HCM. Further research is required to elucidate the mechanisms underlying TTN function.

As the α-heavy chain subunit of cardiac myosin, MYH6 is the fastest molecular motor comprising thick filaments (36). There are close correlations between MYH6 variants and congenital heart defects (CHDs) (37), nonsyndromic coarctation of the aorta (38), familial dilated cardiomyopathy (39), ischemic cardiomyopathy (40), and hypertrophic cardiomyopathy (41). Holm and colleagues found a close correlation between sick sinus syndrome (SSS) and MYH6 missense mutations (42). Thorolfsdottir et al. indicated that atrial fibrillation was associated with rare MYH6 variants, which exerted important effects on the passive elasticity of the heart (43). None of the patients recruited for this study suffered from CHD, coarctation of the aorta, DCM or SSS. The underlying mechanisms by which MYH6 leads to AF remain unclear, and further research is needed.

Ribosomal proteins (RPs) are abundant RNA-binding proteins with multiple functions. One RP, ribosomal protein S27a (RPS27A), actively promotes proliferation in breast cancer (44), renal cancer (45), colon cancer (46), and chronic myeloid leukemia (47). Several studies have revealed the ubiquitination functions of RPS27A. Montellese et al. indicated that USP16-mediated deubiquitination and RPS27a ubiquitination were essential for the entry of maturing pre-40S particles into a pool of translating ribosomes (48). Holm et al. found that P-3F enhances P53 stability by changing the translocation of RPS27a. The release of RPS27a from the nucleolus, from which it enters the nucleoplasm, decreased the phosphorylation of Mdm2 and downregulated the ubiquitination of P53 (49). These reports demonstrated the potential function of RPS27a in translation. A similar mechanism may be involved in AF and needs to be identified.

Accumulated evidence suggests that AF risk is associated with multiomics profiles, including genomics, epigenomics, transcriptomics, proteomics, and metabolomics. Our study found 156 downregulated ubiquitinated proteins and 162 upregulated ubiquitinated proteins in AF patients. In another study based on clinical samples, Amrish Deshmukh et al. revealed 1,011 differentially expressed mRNAs in the LAA tissues between AF and SR patients (50). As Venn diagrams in Supplementary Figures S2A,C show, 8 genes related to downregulated ubiquitinated proteins and increased expression probes simultaneously were obtained in Supplementary Figure S2B, while 8 genes related to upregulated ubiquitinated proteins and decreased expression probes simultaneously were obtained simultaneously in Supplementary Figure S2D. Apart from the proteins mentioned earlier, such as MYH7, MYOM2, MYOM1, and DSP, significant changes in protein ubiquitination levels were observed in FLNC, HSPA1B, and GFPT1 among these intersecting proteins. Filamin C (FLNC) is a protein that plays a vital role in the cytoskeleton structure of cells, and it is highly expressed in cardiac muscle. Numerous studies have suggested a strong correlation between FLNC and the development of cardiomyopathy (51–53). HSPA1B, which encodes heat-shock protein 70 kDa (Hsp70), protects against stroke in AF patients. In our study, HSPA1B was associated with upregulated ubiquitination, and its protection against stroke might be weakened. However, there were no strokes in the enrolled patients (54). Although an increase in ubiquitination level does not necessarily imply protein degradation, combining the results of ubiquitination with results of different -omics layers may help to identify genes that are more relevant to the occurrence and progression of atrial fibrillation.

Multiomics approaches have emerged as a powerful tool with several advantages in studying cardiovascular diseases. One study investigated the association between single-nucleotide polymorphisms (SNPs) and AF by tissue-specific multiomics analysis in a case‒control cohort of AF (55). In their study, genomics, transcriptomics, and proteomics were applied, and correlations between the transcription factor NKX2-5 and AF were elucidated. Multiomics approaches can identify key drivers of disease. As illustrated in the above study, abundant disease-associated SNPs were discovered by genome-wide association studies (GWAS); however, their underlying molecular mechanisms remain largely elusive. With the help of multiomics approaches, the role of NKX2-5 as a link between AF and the GWAS SNP rs9481842 was clarified. The most important advantage of multiomics approaches is that by integrating data from various levels, multiomics approaches can provide a more comprehensive understanding of the molecular mechanisms underlying these complex diseases and the interactions between different biological pathways and networks involved in cardiovascular diseases. Moreover, multiomics approaches can identify new biomarkers and therapeutic targets. Finally, by using multiomics approaches, researchers can develop more precise and personalized approaches to disease diagnosis and treatment. Our study, focused on proteomic changes in the form of ubiquitination, forms an essential piece of this multiomics puzzle. Future studies could use our findings as a stepping stone, integrating them with genomic, transcriptomic, and metabolomic data to fully comprehend the pathogenesis of atrial fibrillation.

Despite being the first study to focus on ubiquitination changes in atrial fibrillation by quantitative proteomics and identifying some valuable proteins with ubiquitination changes, this study still has several limitations. First, because obtaining left atrial appendage tissue during cardiac surgery poses a high risk of perforation and difficulty in obtaining specimens, the sample size was small. Second, current ubiquitin proteomics technology and limited specimen weight per patient may have led to the underrepresentation of ubiquitinated proteins, possibly overlooking some crucial proteins with key functional roles. Third, our center lacks conditions for establishing pig or dog models of atrial fibrillation, precluding functional validation in atrial fibrillation animal models. Finally, differences in amino acid sequences between humans and common animal models used in studying atrial fibrillation (pigs, dogs) may make it difficult to conduct functional studies of some identified proteins using animal models.

In conclusion, significant alterations in ubiquitination were observed between the SR and AF groups. Most DEGs were distributed in ribosome-related proteins with downregulated ubiquitination. The results indicated that alterations in ribosome-associated protein ubiquitination influence the development of AF. Our findings may provide a more feasible strategy against AF.

## Data Availability

The datasets presented in this study can be found in online repositories. The names of the repository/repositories and accession number(s) can be found in the article/**Supplementary Material**.

## Supplementary material

Supplementary Figure S1 Subcellular location of upregulated ubiquitylated proteins (**A**) and downregulated ubiquitylated proteins (**B**).

Supplementary Figure S2 The Venn diagram illustrates the relationship between proteins exhibiting elevated levels of ubiquitination and genes displaying decreased expression (**A**), and the overlapping genes are provided in list form (**B**). Similarly, the Venn diagram depicts the association between proteins exhibiting reduced levels of ubiquitination and genes displaying increased expression (**C**), with the corresponding overlapping genes listed (**D**).
