# Supplementary figures and images for: Changes of ubiquitylated proteins in atrial fibrillation associated with heart valve disease: proteomics in human left atrial appendage tissue
Source: Front Cardiovasc Med. 2023 Aug 28;10:1198486. doi: 10.3389/fcvm.2023.1198486 (PMC10493305; doi:10.3389/fcvm.2023.1198486)

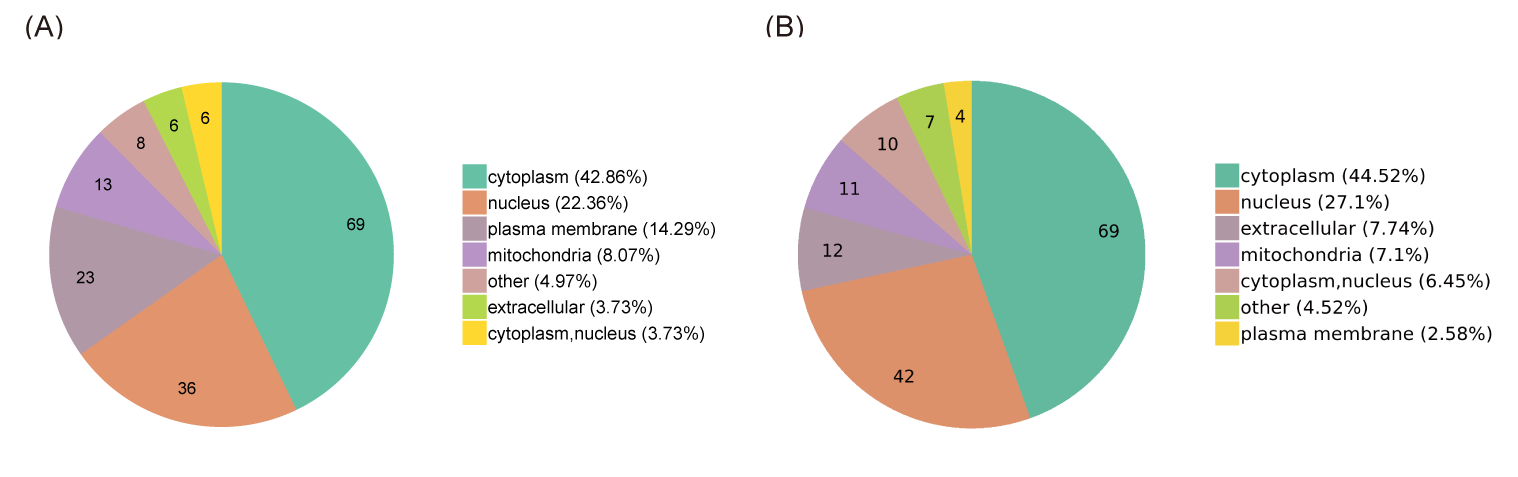

Supplement: Supplementary Figure S1 — Subcellular location of upregulated ubiquitylated proteins (A) and downregulated ubiquitylated proteins (B). [file Image1.tif]

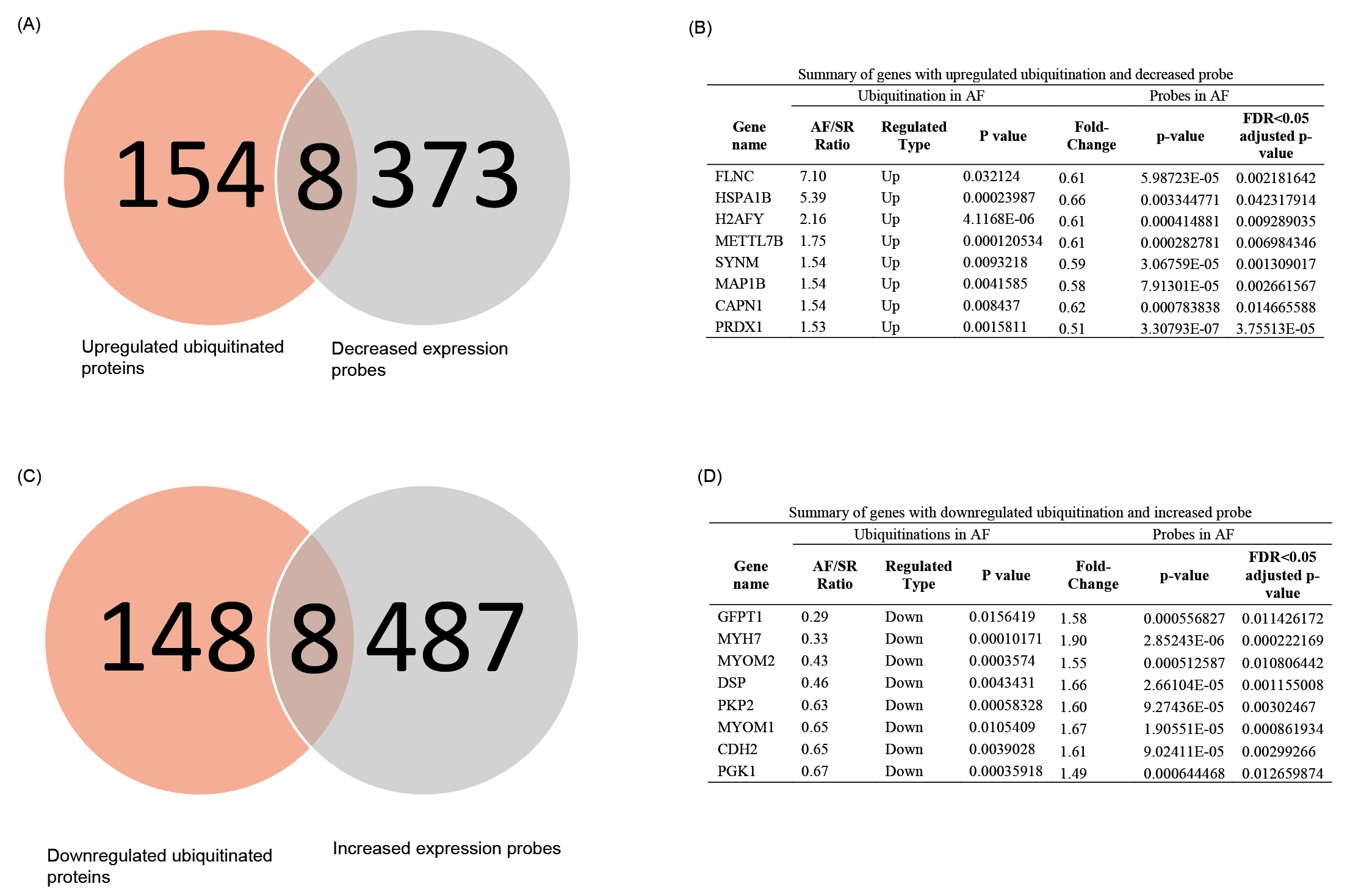

Supplement: Supplementary Figure S2 — The Venn diagram illustrates the relationship between proteins exhibiting elevated levels of ubiquitination and genes displaying decreased expression (A), and the overlapping genes are provided in list form (B). Similarly, the Venn diagram depicts the association between proteins exhibiting reduced levels of ubiquitination and genes displaying increased expression (C), with the corresponding overlapping genes listed (D). [file Image2.tif]
